# Death’s Weapons

**Published:** 1882-03

**Authors:** 


**DEATH'S WEAPONS.**—The higher the state of civilization, the more attention is paid by nations towards ascertaining the causes of disease and death, with kindred subjects, the object being to exercise that parental care and authority which becomes a beneficent government. The glorious English nation is pre-eminent in these regards, our own being too young to have inaugurated any system commensurate with the importance of the subjects. In one year fifty thousand persons died of consumption in England and Wales. It is particularly note-worthy that the mortality from this disease in the city of London bore about the same proportion as that of hilly Wales. And it is not a new remark that, as cities grow older, consumption diminishes; in consequence, no doubt, of the greater intelligence of the people and the greater conveniences and comforts of life. Another fact protrudes itself, and that is, that the doctors do not kill every body. In Wales, twelve of every hundred persons dying had no medical attendant. In one district in England, one person out of every ten had no doctor to help them over the bridge of sighs. Half of all who died were under seventeen years of age. This fearful truth will come more directly home, to parents at least, by saying: "Half of your children will die before entering their eighteenth year!" And why? Because it is natural that they should die thus early? Because they were not made to live longer? Because there is a necessity that it should be so? No; none of these. Nor is it because, inheriting a weakly constitution, they were born diseased. A wise care will overcome these disadvantages in a vast majority of cases. One of the greatest sovereigns in the world was born so decidedly scrofulous as to be threatened with a life-long deformity; and yet, of a houseful of children, not one has died, several have grown up to majority, and all are in high health, and by virtue, too, of a systematic and persistent attention to the laws of hygiene. It clearly follows that half of our children die before they become of age, because they are not properly taken care of, watched over, and instructed as to the means of preserving their health; they are not told by their parents how to avoid disease. This is certainly a fearful reflection, and yet it is undeniably true.

### THE CHERISHED FLOWER.

A FRIEND once presented us with a variety of choice flower seeds, which were duly sown. Among those that came up was a plant of vigorous growth; it always looked fresh and promising, and we concluded it was of unusual value. There was no other one like it, hence we gave it special care. Others not so large began to flower early. But, thinking that early to flower, was early to fade, we gave the pet a still increased attention. The weeds were pulled up from its vicinity as soon as they appeared. The soil was kept in a mellow condition all the time; and, in default of seasonable rain, it was plentifully watered. The attention given to it did not seem to be thrown away; its stalk was strong; the leaves looked fresh and green; but Midsummer had passed, and there was no flower, not even the sign of one. This secured a still increasing care, in the confidence that it was some splendid Fall flower, which would be in its highest prime when those of a common sort had wilted and faded and died away. In proportion as there was no show, pains were taken to protect it from the heats of Summer, and to supply it with the richest manures; its spreading branches fully answered to all the pains bestowed; and no little pleasure was derived in anticipation of feasting the sight on its multitudinous beauties. At last, a flower came, but there was nothing striking about it; in fact, it was insignificant. Our thoughts then took refuge in the surmise that the value of the plant was not in its flower, but in its fruit; that it would yield an ornament for the parlor during the live long Winter, and thus be a beautiful reminder of the Spring time. We waited still, in faith and patience. At long last, the fruition of our hope was in beholding a luxuriant crop of vulgar thistles: all at once, the mind ran after a lesson from it, and a throb of anxiety flitted across our heart, like a black cloud in a Summer's sky. "May be, some child of mine, which I am cherishing with unutterable affection and tenderness, will, after years of ceaseless solicitude, show no promise of that fruitfulness which I have been looking forward to, in sweet illusions, through all the long years of helpless infancy, of dangerous childhood, of wayward youthfulness. Perhaps, that child shall yield no blooming flower, no luscious fruit; the flower and fruit of gratitude, of affectionate watchfulness, of filial deference, of implicit and swift obedience, and, last of all, that tender love, which dissolves itself in tears, when, at my dying hour, I bid a last adieu to all things earthly; but, instead of this, shall be through all my age, and down to the verge of death, only briars.

